# Theaflavins as a novel cross-linker quickly stabilize demineralized dentin collagen against degradation

**DOI:** 10.1038/s41598-021-99186-z

**Published:** 2021-10-05

**Authors:** Hang Liu, Jing Guo, Rong Wang, Yong Wang

**Affiliations:** 1 School of Dentistry, University of Missouri – Kansas City, Kansas City, MO 64108 USA; 2 The Key Laboratory of Oral Biomedicine of Jiangxi Province, and Department of Oral General, the Affiliated Stomatological Hospital of Nanchang University, Nanchang, 330006 China

**Keywords:** Dentistry, Biomedical materials

## Abstract

To investigate the ability of theaflavins (TF) from black tea to protect dentin collagen against enzymatic degradation via cross-linking effect under clinically relevant conditions. 10-µm-thick dentin films were microtomed from dentin slabs of human molars. Following demineralization, films or slabs were treated with TF at two concentrations (0.4% and 2%) for 30 s. A well-known collagen cross-linker grape seed proanthocyanidins (PA) was used as control. Collagen cross-linking interactions and stabilization against enzymatic degradation were investigated by Fourier transform infrared spectroscopy, weight loss, hydroxyproline release, and scanning/transmission electron microscopy. Data were analyzed by ANOVA, Tukey’s and Student’s T test (α = 0.05%). The results showed collagen cross-linking and stabilization efficacy was dependent on TF/PA concentrations. At 2.0%, TF and PA offered nearly full protection to collagen; at 0.4%, TF exhibited a significantly better collagen stabilization effect than PA (*P* < 0.05), while untreated collagen was completely digested. It’s concluded that TF cross-links dentin collagen within a clinically relevant time (30 s) and offers excellent collagen protection against enzymatic degradation, with efficacy comparable to or better than PA. The study supports the potential use of TF as a novel, promising collagen cross-linker for degradation resistant, long-lasting dentin bonding in composite restorations.

## Introduction

Currently, poor durability of dentin bonding is still an immense concern for composite restorations. One of the major reasons is the breakdown of the adhesive/dentin interface over time^1^. More specifically, after acid-etch, the incomplete penetration and curing of the adhesive monomers in the demineralized dentin (DD) surface leave collagen fibers only partially impregnated by the adhesive^2,3^. The unprotected collagen is exposed to oral bacteria such as *Streptococcus mutans*, which excretes organic acids through metabolization^4^. The organic acids (e.g. lactic acid) could activate dormant matrix metalloproteinases (MMPs) in embedded in collagen matrix and lead to collagen degradation^5–7^. In addition, these dormant MMPs are also activated during the acid-etching procedure^8,9^. As a result, enzymatic degradation of unprotected demineralized collagen by MMPs as well as other host-derived enzymes (such as cysteine cathepsins) causes interface breakdown, eventually leading to the failure of the composite restoration^9,10^.

**Figure 1:**
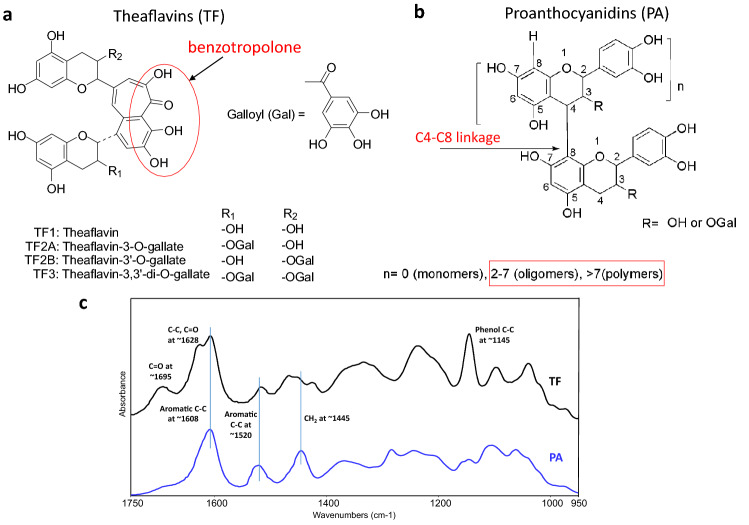
Schematic diagrams of molecular structures of theaflavins (TF) from black tea (**a**) and proanthocyanidins (PA) from grape seed (**b**); representative FTIR spectra of TF and PA powders (**c**).

To solve this degradation issue, collagen cross-linkers have been applied to the interface and their protection efficacies have been studied^11,12^. A good collagen cross-linker should have strong intermolecular interactions with multiple collagen fibrils, and as a result, enhance the structural stability of the collagen matrix^13,14^. The cross-linker stabilized dentin collagen becomes more resistant to enzymatic degradation. It is reported that some bi-functional aldehydes, e.g. glutaraldehyde, originally applied in the tanning industry^15^, could react with collagen and form strong chemical bonds within collagen fibrils^16^. However, the effort to introduce these chemicals into oral environments can hardly succeed because of their cytotoxicity^17,18^ and long reaction time^19^. Due to these concerns, botanical extracts containing natural polyphenols have drawn a lot of attention in dental research. Among all the natural extracts studied, proanthocyanidins (PA) from the grape seed proves to be a highly efficient collagen cross-linker due to its low toxicity^20^, high effectiveness and rapidness^21^ in cross-linking dentin collagen. Previously we reported that PA was capable of cross-linking demineralized dentin (DD) layer within a clinically relevant time of 15–30 s^21,22^, which is very important since longer treatment time (e.g. minutes) is generally not feasible in clinical settings where patients are treated with mouth open and the procedure time plays an important role in the quality of restorations and patient experience. The DD layer treated by PA for 30 s becomes very stable and degradation resistant to an extremely high concentration of MMP-1 (collagenase I). However, PA is still far from an ideal collagen cross-linker. For instance, the molecular constitution of PA is very complicated (Fig. 1b). Although the basic building blocks of PA are monomeric catechins, which form PA by oxidative coupling with each other, PA actually is a mixture of dimers, trimers, tetramers, and much higher molecular weight (MW) polymers of catechins^23^. Therefore, it is very difficult to find out a clear relationship between specific chemical structure and the crosslinking property from the studies of PA because of the difficulty in separating each compound from PA, especially the high MW species, which are believed to be more effective than lower MW species in collagen interactions^24^. Moreover, the large size of the high MW PA species might limit their effectiveness due to slow/poor penetration into the DD layer, especially at a low concentration^22,25^. A low concentration of PA is preferred in clinical settings because excess non-reacted PA may hamper the curing of adhesives through a radical scavenging effect^11,12^. In light of this, there is still an ongoing endeavor to look for a better natural collagen cross-linker for dental applications.

Tea extracts contain an abundance of polyphenols as well such as catechins. As the basic building blocks of polyphenols, monomeric catechins have been previously investigated for their collagen cross-linking capability. Among all the monomeric catechins, ( −)-epigallocatechin-3-gallate (EGCG) and ( −)-epigallocatechin (EGC) from green tea extract were found to be most effective, which showed a significant correlation with the galloyl moieties^14^. However, although the degradation resistance of dentin collagen could be improved by EGCG or EGC, their effects were not as good as PA (less than 50% protection)^24^. Besides monomeric catechins, another type of polyphenols, theaflavins (TF), is also abundant in tea extracts, especially from black tea (Camellia sinensis). TF is found to have multiple bioactivities including anti-bacterial^26^, anti-HIV^27^, anti-cancer^28^, cholesterol-lowering^29^ as well as low toxicity to normal cells^30^. These bioactivities are due to the intermolecular interactions between TF and various proteins, including some receptors^31,32^. As compared to PA, the molecular constitution of TF is less complicated, contains four dominant structures of dimers (Fig. 1a), which are formed by oxidative coupling of two monomeric catechin molecules. Different from PA dimers in which two catechins are connected via a single C–C bond, the two catechin units in TF are coupled through a benzotropolone skeleton (Fig. 1a). This skeleton structure contains a carbonyl group at the adjacent position of a hydroxyl group. The carbonyl group has the potential of forming hydrogen bonding with a hydrogen donor, e.g. hydroxyl or amine group, which may favor collagen cross-linking. From the chemical structure aspect, TF has aromatic rings attached with abundant phenol hydroxyl groups as well as galloyl groups. According to previous studies, these structures play critical roles in collagen cross-linking^33–35^. The aim of this study was to investigate the effects of TF on collagen cross-linking and resistance to enzymatic degradation. The null hypotheses tested were that TF would not be able to (1) cross-link dentin collagen via chemical interactions or (2) stabilize dentin collagen against enzymatic degradation.

## Results

### FTIR characterization of TF and PA

Representative FTIR spectra of TF and PA powders are shown in Fig. 1c. Characteristic bands associated with PA powder were identified and assigned to vibrations of different functional groups as well documented in the literature, such as the phenyl ring backbone C–C stretching vibrations at ∼1608 cm^−1^ and ∼1520 cm^−1^, the CH_2_ bending vibration at ~ 1445 cm^−1^, and the O–H bending, ether, phenol C–C stretching vibrations between 1400 cm^−1^ and 1000 cm^−1^. The FTIR spectrum of TF was quite similar to that of PA, except for the bands at ∼1695 cm^−1^, ∼1628 cm^−1^, and ∼1145 cm^−1^, which represent the unique C = O stretching, backbone C–C stretching, and phenol C–C stretching of the benzotropolone moiety, respectively (Fig. 1a). The MWs of the four TF structures are 564.5 (TF1), 716.6 (TF2A), 716.6 (TF2B), 868.7 (TF3), respectively, which are close to those of PA dimers. However, PA’s MW range is much larger since it also contains higher MW species, e.g. tetramers, pentamers…polymers (range from monomer to polymer n > 7) (Fig. 1b).

### FTIR spectra of demineralized dentin films treated with TF or PA at different concentrations

**Figure 2:**
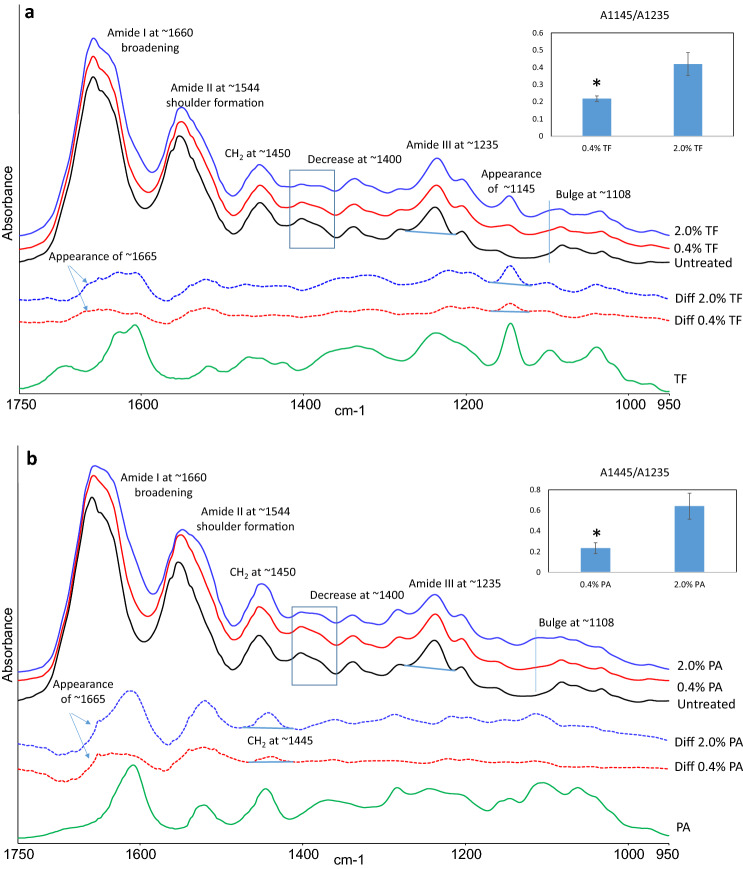
(**a**) Representative FTIR spectra of dentin collagen before (black line) and after the treatment of either 0.4% TF (red line) or 2.0% TF (blue line) for 30 s. Difference spectra of treated collagen films with the spectrum of untreated collagen subtracted (dashed lines) are shown in the middle and the spectrum of TF powder (green line) is shown in the bottom for comparison. Inset: the band ratios of A1145/A1235 for 0.4% TF and 2.0% TF treated collagen (*P < 0.001, n = 6). (**b**) Representative FTIR spectra of dentin collagen before (black line) and after the treatment of either 0.4% PA (red line) or 2.0% PA (blue line) for 30 s. Difference spectra of treated collagen films with the spectrum of untreated collagen subtracted (dashed lines) are shown in the middle and the spectrum of PA powder (green line) is shown in the bottom for comparison. Inset: the band ratios of A1445/A1235 for 0.4% PA and 2.0% PA treated collagen (*P < 0.001, n = 6).

Representative FTIR spectra of untreated collagen, collagen treated with TF or PA for 30 s at different concentrations are shown in Fig. 2. The characteristic bands related to collagen were assigned, such as C=O stretching at ~ 1660 cm^−1^for amide I, out-of-phase combination of N–H bending and C-N stretching at ~ 1544 cm^−1^for amide II, CH_2_ bending at ~ 1450 cm^−1^, and in-phase combination of N–H bending and C-N stretching at ~ 1235 cm^−1^ for amide III. Although some spectral bands from TF and PA (shown in the bottom of Fig. 2 for comparison) overlapped with those from collagen, there were still noticeable spectral changes when comparing the treated with untreated collagen. For example, bulge formation at ~ 1108 cm^−1^, decrease in intensity at ~ 1400 cm^−1^, right shoulder formation of amide II (~ 1544 cm^−1^), and the broadening of amide I (~ 1660 cm^−1^) to lower wavenumbers were evidently identified after only 30 s treatment of TF or PA at both 0.4% and 2.0% concentrations. In addition, a sharp band at ~ 1145 cm^−1^ appeared in the spectra of TF-treated dentin collagen. A closer glance showed that the extent of the above changes was dependent on the concentration of crosslinkers used. When comparing to the untreated control, more marked changes were detected at 2.0% concentration, while less changes were identified at 0.4% concentration.

To further look into the potential collagen crosslinking interactions with different concentrations of crosslinkers, the spectrum of untreated collagen was subtracted from the spectra of collagen specimens that were treated with TF or PA at different concentrations. Using the amide III band as the internal standard for subtraction, the difference spectra are shown in Fig. 2 along with the spectrum of TF or PA powders. Most of the bands in the difference spectra could find matching counterparts in the spectrum of TF or PA powder, except for one broad shoulder band at ~ 1665 cm^−1^. The ratio of the band either at 1145 cm^−1^ (for TF) or 1445 cm^−1^ (for PA) to amide III band at 1235 cm^−1^ was used to quantify the extent of interactions between collagen and crosslinker (Fig. 2 insets). The ratios for collagen films treated with 2% TF or PA were significantly higher than those treated with 0.4% crosslinker respectively (*P* < 0.001). Specifically, the ratio (A1145/A1235) at 0.4% TF (0.22 ± 0.02) was about half of the ratio at 2.0% TF (0.42 ± 0.07), while the ratio (A1445/A1235) at 0.4% PA (0.23 ± 0.05) was about one-third of the ratio at 2.0% PA (0.64 ± 0.13).

### Weight loss and HYP release of cross-linked dentin collagen films after collagenase digestion

**Figure 3:**
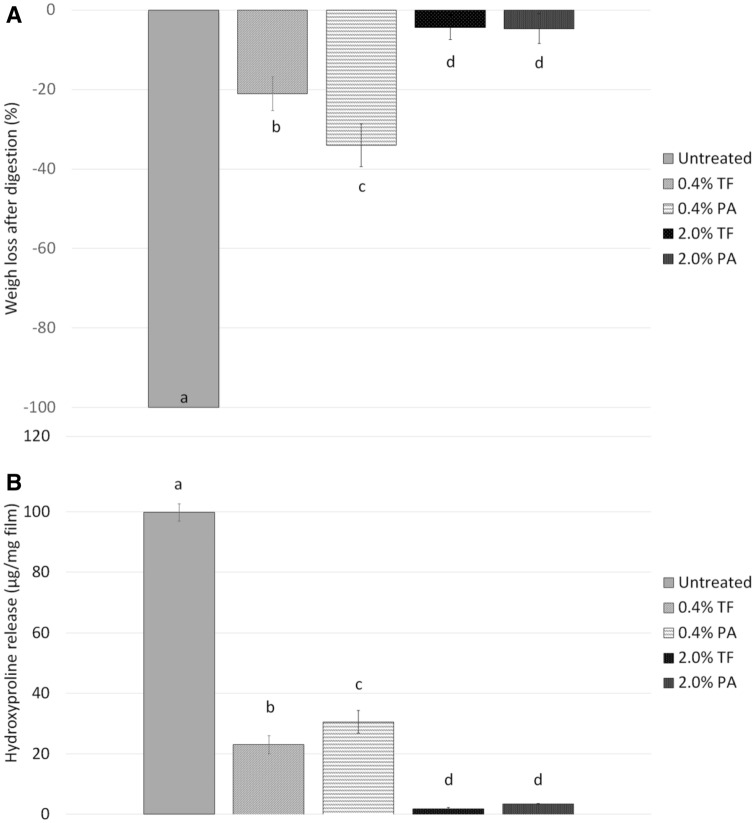
Means and standard-deviations of dentin collagen resistance against collagenase digestion measured by (**A**) weight loss (%) and (**B**) hydroxyproline proline release (µg/mg film). Different lowercase letters demonstrate statistically significant differences among groups (n = 6, *P* < 0.05).

Figure 3A shows the weight change of the demineralized dentin films after collagenase digestion. The untreated films (control) completely degraded within 1 h of digestion. The films visually disappeared in the digestant solution with no residue left. For this group, the average amount of HYP released into the solution from each milligram of the films was 99.8 (± 2.8) µg/mg (Fig. 3B), which was approximately all the hydroxyproline contained in the tested dentin collagen (mainly type I collagen with 10 wt% of hydroxyproline). At 2.0% crosslinker concentration, both TF and PA treatments made dentin collagen almost completely resistant to collagenase degradation, with an average WL of 4.3% and HYP release of 1.8 µg/mg for TF, and an average WL of 4.7% and HYP release of 3.4 µg/mg for PA treatment. At 0.4% crosslinker concentration, the average WL and HYP were 34.0% and 30.6 µg/mg for PA treatment, which were significantly higher (*P* < 0.05) than the average WL and HYP of 21.0% and 23.0 ug/mg for TF treatment, respectively.

### Scanning electron microscopy (SEM)

**Figure 4:**
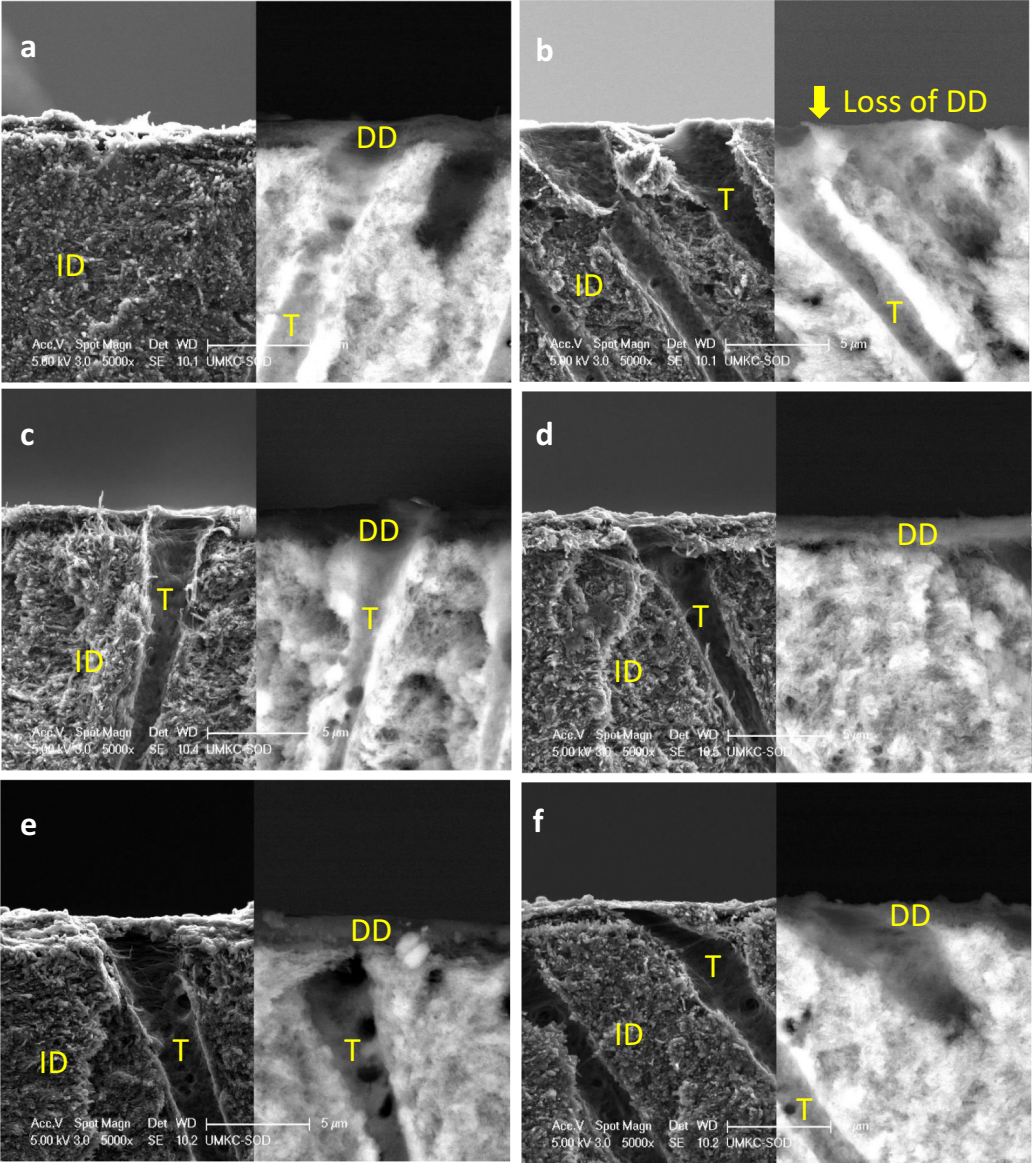
Secondary-electron (SE, left) and back-scattered electron (BSE, right) SEM images of acid-etched dentin without treatment (**a**) before and (**b**) after digestion (in 0.1% collagenase at 37 °C for 1 h); treated with 2.0% TF for 30 s followed by digestion (**c**); treated with 2.0% PA for 30 s followed by digestion (**d**); treated with 0.4% TF for 30 s followed by digestion (**e**); and treated with 0.4% PA for 30 s followed by digestion (**f**). The demineralized dentin layer of untreated control was evident as dark layer atop intact dentin under BSE mode before digestion (**a**), and was completely lost after digestion (**b**), while that of all treated groups (**c**–**f**) remained after digestion. *DD* demineralized dentin. *ID* intact dentin. *T* tubule.

Figure 4 shows secondary-electron (SE, left) and back-scattered electron (BSE, right) SEM images of fractured cross-sections of acid-etched dentin slabs w/wo crosslinking treatments. When not challenged by collagenase, a thin demineralized dentin (DD) layer characterized by dark color under BSE mode (Fig. 4a) was clearly visible. After digestion in 0.1% collagenase at 37 °C for 1 h, the DD layer of the untreated control was completely gone (Fig. 4b), while the DD layers treated with TF or PA remained unchanged in both morphology and thickness after digestion, regardless of the treatment concentration (0.4% or 2.0%) (Fig. 4c–f), indicating resistance to degradation.

### Transmission electron microscopy (TEM)

**Figure 5:**
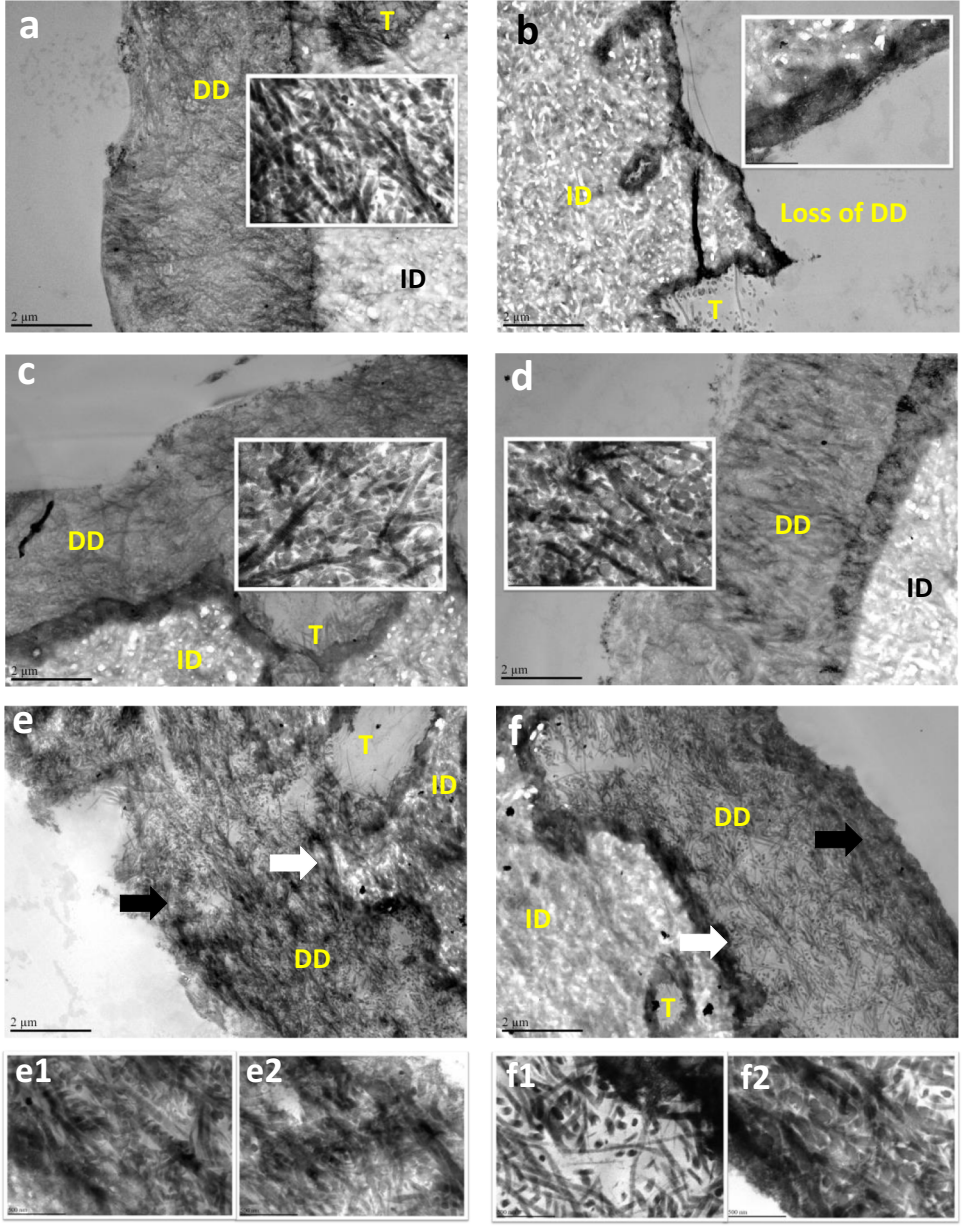
TEM images of acid-etched dentin without treatment (**a**) before and (**b**) after digestion (in 0.1% collagenase at 37 °C for 1 h); treated with 2.0% TF for 30 s followed by digestion (**c**); treated with 2.0% PA for 30 s followed by digestion (**d**); treated with 0.4% TF for 30 s followed by digestion (**e**); and treated with 0.4% PA for 30 s followed digestion (**f**). Representative high magnification view of the demineralized dentin (DD) layer shown as inset (**a**–**d**); high magnification view of the bottom region (white arrow position) in DD layer (**e1** and **f1**); high magnification view of the top region (black arrow position) in DD layer (**e2** and **f2**). DD: demineralized dentin. *ID* intact dentin. *T* tubule.

The TEM images of undigested acid-etched dentin slabs displayed an obvious DD layer (Fig. 5a), in which collagen fibers exhibited the characteristic 67-nm banding pattern (inset). For the untreated control, this layer completely degraded after digestion (Fig. 5b). At 2.0% crosslinker concentration, both TF and PA treatment groups kept good integrity of the DD layer and the characteristic pattern of collagen fibers after digestion (Fig. 5c,d). When the crosslinker concentration was reduced to 0.4%, both TF (Fig. 5e) and PA (Fig. 5f) treatment groups still maintained the DD layer. At the higher magnification, the DD layer treated with 0.4% TF was not affected by digestion, either in the bottom (Fig. 5e1) or top region (Fig. 5e2); however, the DD layer treated with 0.4% PA showed lower density of collagen fibers in the bottom region (Fig. 5f1) than that in the top region (Fig. 5f2) after digestion, indicating partial degradation of the collagen matrix.

## Discussion

Over the past years, theaflavins (TF) have drawn so much attention due to their many bioactivities such as anti-cancer, anti-microbial and anti-inflammatory effects. In dentistry, TF has shown inhibition of *S. mutans*, anti-caries function^36,37^ and beneficial effects against periodontal disease^32,38^. TF has been reported to be the main polyphenols responsible for astringency and sensation of black tea. Astringency is a tangible taste felt as a dry feeling in the mouth and contraction of the tongue tissue, which generally implicates the instantaneous binding between polyphenols and proline-rich proteins in the saliva. It is well known that collagen is also rich in prolines, which could potentially interact with TF in similar ways. The multiple phenol hydroxyl groups and galloyl moieties in the TF structure make it a potential crosslinker for collagen. However, TF has never been studied in its collagen crosslinking function to date. The current study was therefore carried out to investigate the ability of TF to protect dentin collagen from collagenase degradation via cross-linking within a clinically relevant time.

In our investigation, 10-um-thick demineralized dentin films were used to simulate the acid-etched, demineralized dentin layer where collagen degradation usually occurs. The films were treated with different concentrations of TF or PA for 30 s. FTIR spectra revealed that most of the spectral changes of dentin collagen caused by TF treatment were comparable to those by PA treatment, as shown in Fig. 2. One of the most predominant changes was the decreased intensity of the band at ~ 1400 cm^−1^, which was caused by the dehydration of collagen fibers, an important evidence of cross-linking^33–35^. The collagen dehydration was generated by the substitution of bound water in collagen by TF/PA, which has stronger hydrogen bonding interactions with collagen than water. The broadening of amide I (~ 1660 cm^−1^) and shoulder formation of amide II (~ 1544 cm^−1^) observed in Fig. 2 are also attributed to hydrogen bonding via amino and amide groups of collagen and phenolic hydroxyl (OH) groups of TF/PA^39,40^.

The above spectral changes were generally more pronounced in the higher concentration (2%) treatment groups than in the lower concentration treatment groups (0.4%). Subtracting the untreated collagen spectrum from the spectra of the treated collagen could bring out the changes due to interactions with TF/PA. The resultant difference spectra (Fig. 2) further verified the incorporation of TF/PA in collagen. The band ratio of A1145/A1235 or A1445/A1235 was used to quantify the extent of TF or PA interaction/incorporation with collagen, respectively (Fig. 2 insets). The higher the ratio, the stronger the interaction between collagen and the crosslinker of either TF or PA. The results showed significantly higher ratios for the 2% groups than for the 0.4% groups (*P* < 0.001). Most of the bands in the difference spectra can be matched to the spectra of the corresponding crosslinker powders (Fig. 2), including the band at ∼1145 cm^−1^, which is assigned to the C–C(-OH) stretching on the benzotropolone of TF (Fig. 1a). There was a new broad shoulder band at ~ 1665 cm^−1^ that could not be detected in the crosslinker powders. This new band indicates another interaction of covalent bonding^41,42^, which has been attributed to the imine (C=N) stretching of the Schiff base formed between PA component and collagen^33–35^. Such covalent bond formation involves auto-oxidation of catechol moieties to ortho-quinone groups, which subsequently react with free amine groups on collagen^43^. This shoulder band also emerged in TF-treated collagen, indicating similar covalent bond formation. All the results indicate strong crosslinking interactions of TF with dentin collagen. Therefore, the first null hypothesis was rejected.

Cross-linking could alter the mechanical and physicochemical properties of dentin collagen. Compared to enhancement in mechanical strength of dentin collagen which usually needs longer treatment times (more than 10 min)^44–46^, it is of more interest to see if dentin collagen could become resistant to degradation within clinically relevant time (30 s) after cross-linking^21^. To quantitatively evaluate the degradation resistance of TF-treated dentin collagen, the WL and HYP release of the treated collagen films were measured after challenged by 0.1% collagenase (MMP-1) solution to simulate the enzyme-induced degradation of dentin collagen. Both WL and HYP results indicated that TF-treated films developed strong resistance to enzymatic degradation and the degradation resistance was related to the concentration of TF (Fig. 3). At 2.0% concentration, the TF-treated films became almost immune to digestion; and the PA-treated films showed similar protection, which is consistent with previous PA results^22^. At 0.4% concentration, both the WL and HYP release values of the TF-treated films were significantly lower than those of the PA-treated films, indicating TF is even more effective in stabilizing dentin collagen than PA. This is in agreement with the FTIR results, which showed that the ratio (A1145/A1235) at 0.4% TF was about half of that at 2.0% TF, while the ratio (A1445/A1235) at 0.4% PA was only about one-third of that at 2.0% PA. It is worth pointing out that the concentration of collagenase (0.1%) used in the test was 2–6 orders of magnitude higher than the MMP level found in human oral fluids^47,48^. In comparison the untreated films (control) completely degraded within 1 h of digestion. Those results provide strong evidence to support the ability of TF in stabilizing dentin collagen against enzymatic degradation. Therefore, the second null hypothesis was rejected.

It would be beneficial to test the performance of TF on the acid-etched layer of dentin bonding substrates. To mimic the clinical condition, the surface of a dentin slab was etched by 35% phosphoric acid gel for 15 s to generate a demineralized layer, treated with TF/PA for 30 s as a primer, and then subject to 0.1% collagenase degradation for 1 h. According to the SEM images (Fig. 4), TF could rapidly (30 s) cross-link the acid-etched layer of dentin and protect the demineralized collagen matrix from degradation even at a very low concentration of 0.4%. This is very encouraging since a low concentration of crosslinker is preferred in clinical conditions to avoid possible adverse effects (e.g. staining, polymerization inhibition)^11^ of free cross-linkers left in collagen matrix due to inadequate rinse.

The detailed structure/morphology of the acid etched DD layer could be observed by TEM. At a low magnification, the TEM images of the DD layer (Fig. 5a–f) were consistent with corresponding SE/BSE SEM images (Fig. 4). The acid-etched dentin layers primed with TF/PA solutions maintained their integrity after digestion, regardless of the treatment concentrations used. At a higher magnification, the TEM images showed that the internal structure/morphology of TF-treated DD layers was not affected by digestion at both concentrations (Fig. 5c,e). In comparison, PA treated DD layers showed good integrity only at 2.0% concentration. The collagen fibers at the bottom of the DD layer primed with 0.4%PA were partially lost, indicated by the decreased density of collagen fibers (Fig. 5f1), while the surface region of this layer was not significantly affected (Fig. 5f2). The different performance of TF and PA at 0.4% concentration may be caused by their difference in crosslinking efficiency and/or by high MWs of PA. During priming, the cross-linkers penetrate into the DD layer via diffusion. As a result, there should be a concentration gradient between the surface and the bottom regions. In general, the larger the molecule, the more slowly it penetrates, and the greater the concentration gradient will be. The sizes of the four TF molecules are similar to that of a PA dimer, much smaller than the high MW PA molecules (Fig. 1). Therefore, smaller TF molecules may penetrate much faster into the DD layer than the high MW PA molecules. Since the high MW molecules of PA contribute more to collagen stabilization than the low MW PA molecules^24^, it may help explain why TF could protect the bottom of the DD layer better than PA at a low concentration.

The above results indicated that TF is an excellent collagen crosslinker, even better than PA at low concentrations. This could be attributed to the unique extra fused ring of benzotropolone and/or a large number of phenolic OH groups in TF. Different from the complex molecular constitutions of grape seed PA which are hard to purify/separate, TF has a much simpler constitution. As shown in Fig. 1, TF mainly consists of four compounds, theaflavin (TF1), theaflavin-3-gallate (TF2A), theaflavin-3’-gallate (TF2B), and theaflavin-3,3’-digallate (TF3), which differ in the number and position of the linked galloyl moiety: TF1 has no galloyl group; TF2A and TF2B contain a galloyl group to replace one hydroxyl group of TF1 at two different positions; and TF3 has two galloyl groups. It would be interesting to know the potential function of benzotropolone in TF and which one of the four TF compounds is a better crosslinker individually. For example, a previous study indicated that the PA dimer (epicatechin-epicatechin, EC-EC) with no galloyl group did not show any collagen crosslinking and protection effects^24^. It would be of interest to see if TF1, similar to EC-EC with the exception of the benzotropolone connection, could induce collagen crosslinking. It would also be of interest to explore how the number and position of the phenolic hydroxyl and galloyl groups in TFs1-3 affect their interactions with collagen. The structure–activity relationship of the four TF compounds extracted from black tea in their collagen stabilization function is the topic of our next investigation.

## Conclusion

In this study, we demonstrated that TF from black tea extract could rapidly cross-link demineralized dentin collagen and protect it from collagenase degradation under clinically relevant conditions. To the best of our knowledge, this is the first study to evaluate the crosslinking and stabilization effects of TF on collagen. Our findings show that the protective efficiency of TF is similar to or even higher than that of the highly efficient PA depending on the concentrations. The promising results warrant further investigations of the structure–activity relationship of TF in dentin collagen crosslinking and clinical applications of TF in restorative dentistry.

## Materials and methods

### Reagents

TF (theaflavins, from Camellia sinensis) and collagenase type I (from Clostridium histolyticum, 125 U/ mg) were purchased from Sigma-Aldrich (St. Louis, MO, USA). PA (proanthocyanidins, from grape seed) was donated by the manufacturer (Polyphenolics, Madera, CA, USA). 0.96% phosphate buffered saline (PBS, pH = 7.4) was prepared using Sigma Life Science—Dulbeccos Phosphate Buffered Saline packet, 0.002% sodium azide was added to prevent bacterial growth. TESCA buffer was prepared by dissolving 5.75 g of TES (N-tris (hydroxymethyl) methyl-2-aminoethanesulfonic acid, Lot 103181, Fisher Scientific, Pittsburgh, PA, USA), and 26.5 mg of CaCl_2_ (Lot 876772, Fisher Scientific) in 500 mL of distilled water, and the pH was adjusted to 7.4 using NaOH.

### Demineralized dentin films preparation and cross-linking treatment

Non-carious human molars were collected with no associated patient identifiers, collection protocol determined as not human subject research (NHSR 12–50) as per the Adult Health Sciences Institutional Review Board (IRB) of the University of Missouri Kansas City. All experimental protocols were approved by the University Institutional Biosafety Committee (IBC) and all methods were carried out in accordance with relevant guidelines and regulations. Extracted teeth were stored at 4 °C in 0.96% (w/v) phosphate-buffered saline containing 0.002% sodium azide. The occlusal portion of the crown and side enamel walls were removed from randomly selected teeth by a water-cooled low-speed diamond saw (Buehler, Lake Bluff, IL, USA). The resultant dentin blocks were sectioned into 10-μm-thick films in the mesial-distal direction with a tungsten carbide knife mounted on the SM2500S microtome (Leica, Deerfield, IL, USA). Approximately fifty-five films were obtained from each tooth in a size of approximately 6 × 5 mm, which resulted in a total of 330 dentin films from six molars. These films were randomly assigned into 5 groups (n = 66/group) according to the concentration of crosslinker treatment: 0.4% TF, 2.0% TF, 0.4% PA, 2.0% PA (all in 90/10 (v/v) ethanol/PBS) and an untreated group as a negative control (solvent only). Each dentin film was demineralized with 10% H_3_PO_4_ for 30 min, rinsed with deionized (DI) water for 10 s, and spread by a fine paintbrush onto a plastic cover slip (Fisher Scientific). After blotting away the excess water, each of the demineralized films was completely immersed in a drop (~ 30 uL) of the selected treatment solution. After 30 s, the films were rinsed with DI water for 30 min (changing DI water every 10 min), then dried for 48 h in a desiccator under vacuum. The treated and dried films were subsequently analyzed by FTIR (n = 6 per treatment group) and evaluated for collagen stability via weight loss (WL) and hydroxyproline release assay (HYP) (n = 60 per group).

### FIIR analysis of demineralized dentin films

The six treated and dried dentin collagen films from each group were submitted to Fourier-transform infrared spectroscopy (FTIR) examination (Spectrum One, Perkin Elmer, Waltham, MA, USA) on a BaF_2_ disc, to analyze collagen crosslinking effect of TF and PA via chemical interaction. Each FTIR spectrum was collected in the 1750–950 cm^−1^ wavenumber range at a resolution of 4 cm^−1^ and a scan number of 64. For spectral analysis, the spectrum of untreated dentin collagen film was subtracted from the spectra of treated collagen films to clarify the interactions between the collagen and cross-linkers. The integration areas of the absorbance band at either 1145 cm^−1^ (for TF) or 1445 cm^−1^ (for PA) of the resultant difference spectra were measured to demonstrate the degree of chemical interactions with collagen, while the integration band area at 1235 cm^−1^ (amide III) of untreated collagen was utilized as an internal reference. Then, the band ratio of either A1145/A1235 or A1445/A1235 was calculated for each of TF or PA treatment groups, respectively.

### Weight loss and hydroxyproline release of demineralized dentin films challenged by collagenase digestion

The 60 treated dentin collagen films per treatment group were randomly distributed into 6 sub-groups including 10 films in each specimen group. The films were submerged in 300 µL of 0.1% bacterial collagenase solution in TESCA at 37 °C for 1 h. Following the rinse with DI water for 30 min (changing DI water every 10 min) and drying for 48 h (in a desiccator), the WL percentage was calculated by the weight change of dried films before (W_0_) and after (W_D_) collagenase digestion for each specimen measured by an analytical balance (d = 0.01 mg, Mettler Toledo AG285, Zurich, Switzerland), and was determined by the following equation: WL% = ((W_0_-W_D_)/W_0_) × 100%.

For the HYP assay, the detailed protocol could be found in the previous publication^49^. Briefly, the digestion solution was gathered and hydrolyzed at 110 °C in 6 M HCl for 24 h and freeze-dried. The dry remainder (free hydroxyproline and other amino acids) from each specimen was first treated by neutralization, followed by oxidation, and then subjected to 5% Ehrlich’s reagent (5% dimethylamino benzaldehyde dissolved in n-propanol) to develop the color. The absorbance was measured with a microplate reader (Biotek Instruments, Winooski, VT, USA) at 555 nm. The trans-4-hydroxy-L-proline (analytical standard, Sigma-Aldrich) was utilized as the standard to acquire the working curve for computing the HYP release (n = 6) from each micro gram of demineralized collagen films during the digestion.

### Preparation, acid etching and crosslinking of dentin slabs

Non-carious human molars were sectioned into dentin slabs as follows. After removing crown, a uniform smear layer was produced on the dentin surface using wet 600-grit SiC sandpaper (Buehler) for 30 s. Further sections were generated in the occlusal-apical direction at increments of 2 mm (for SEM) or 0.5 mm (for TEM), followed by one cut parallel to and either ~ 1.5 mm (for SEM) or 0.5 mm (for TEM) below the abraded surface to free the slabs. Slabs for SEM were notched at the center position from the side opposite to the abraded surface, for subsequent fracturing to permit visualization of the dentin subsurface.

The abraded surfaces of the slabs were etched for 15 s with 35% phosphoric acid gel (Scotchbond Etchant, 3 M-ESPE, St. Paul, MN, USA) and rinsed with water for 10 s. After being blot-dried, the slabs were pooled, randomly selected, and treated with either TF (0.4%, 2.0%) or PA (0.4%, 2.0%) in 90/10 (v/v) ethanol/PBS for 30 s. Upon completion of treatment, the slabs were immediately submerged in a copious amount of de-ionized water and then rinsed for an additional 30 min (with the water changed every 10 min). Slabs that received no crosslinker treatment (only with the above solvent for 30 s) served as negative control. For each group, at least 8 slabs were prepared, half of which were not subject to collagenase digestion, whereas the other half underwent 1 h of digestion, at 37 °C in 1 mL of 0.1% collagenase solution.

### Scanning electron microscopy (SEM)

The notched slabs were fixed for 1 h in 2.5% glutaraldehyde buffered with 0.1 M sodium cacodylate, and dehydrated using graded solutions of ethanol (33%, 67%, 85%, 95% and 100%) for 2 h at each concentration. After being air-dried overnight, the slabs were fractured, mounted on aluminum stubs with conductive tape, and coated with carbon. The fractured cross-sections were then visualized using an FEI/Philips XL30 Field-Emission Environmental SEM (Philips, Eindhoven, Netherlands) in the secondary electron mode (SE) and back-scattered electron mode (BSE).

### Transmission electron microscopy (TEM)

The un-notched slabs were post-fixed with 1% osmium tetroxide for 1.5 h and dehydrated in graded solutions of ethanol as described earlier. Then the specimens were treated with 1:1 solution of ethanol and propylene oxide for 30 min, followed with 100% propylene oxide for 2 h, and finally with 1:1 solution of propylene oxide and epoxy resin (Embed-812, Electron Microscopy Sciences) overnight. After final infiltration with pure epoxy resin, specimens were incubated in an oven at 60 °C for 48 h. Ultrathin (100 nm) sections were cut with an EM-UC7 ultramicrotome (Leica, Buffalo Grove, IL, USA), stained with 1% phosphotungstic acid and 2% uranyl acetate, and observed with a CM12 electron microscope (FEI, Hillsboro, OR, USA) at 80 kV accelerating voltage.

### Statistical analysis

The differences in FTIR ratios between the two concentrations of treatment solutions were analyzed using Student’s t test (α = 0.05). Data from weight loss, and hydroxyproline content were evaluated using one-way analysis of variance (ANOVA), followed by Tukey post-hoc test (α = 0.05). Statistical analysis was performed using GraphPad Prism 5 (GraphPad Software, San Diego, California, U.S.).
